# Contribution of microglia/macrophage to the pathogenesis of TMEV infection in the central nervous system

**DOI:** 10.3389/fmicb.2024.1452390

**Published:** 2024-08-02

**Authors:** Qianye Zhang, Wei Sun, Mingxiao Zheng, Ning Zhang

**Affiliations:** Institute of Biopharmaceutical Research, Liaocheng University, Liaocheng, Shandong, China

**Keywords:** Theiler’s murine encephalomyelitis virus (TMEV), multiple sclerosis (MS), microglia/macrophage, M1/M2, neuroinflammation

## Abstract

The infection of the central nervous system (CNS) with neurotropic viruses induces neuroinflammation and an immune response, which is associated with the development of neuroinflammatory and neurodegenerative diseases, including multiple sclerosis (MS). The activation of both innate and adaptive immune responses, involving microglia, macrophages, and T and B cells, while required for efficient viral control within the CNS, is also associated with neuropathology. Under pathological events, such as CNS viral infection, microglia/macrophage undergo a reactive response, leading to the infiltration of immune cells from the periphery into the brain, disrupting CNS homeostasis and contributing to the pathogenesis of disease. The Theiler’s murine encephalomyelitis virus (TMEV)-induced demyelination disease (TMEV-IDD), which serves as a mouse model of MS. This murine model made significant contributions to our understanding of the pathophysiology of MS following subsequent to infection. Microglia/macrophages could be activated into two different states, classic activated state (M1 state) and alternative activated state (M2 state) during TMEV infection. M1 possesses the capacity to initiate inflammatory response and secretes pro-inflammatory cytokines, and M2-liked microglia/macrophages are anti-inflammatory characterized by the secretion of anti-inflammatory cytokines. This review aims to discuss the roles of microglia/macrophages M1/M2-liked polarization during TMEV infection, and explore the potential therapeutic effect of balancing M1/M2-liked polarization of microglia/macrophages on MS.

## 1 Introduction

Theiler’s murine encephalomyelitis virus (TMEV) belongs to the genus cardiovirus of the picornaviridae family (Gerhauser et al., 2019). The pathogenesis of TMEV involves a complex interaction between viral infection and the host immune response, particularly the activation of glial cells and the immune response to the central nervous system (CNS) (DePaula-Silva et al., 2021). Microglia and macrophagesare highly engaged in the neuroinflammatory process during viral encephalitis and are key contributors to the initiation of the innate and adaptive immune response (Bosco et al., 2020; Filgueira et al., 2021). TMEV-induced demyelinating disease (TMEV-IDD) represents a well-established animal model for demyelinating diseases in humans, especially resembling significant characteristics of the progressive forms of MS (Pike et al., 2022). This review will focus on the role of microglia and infiltrating peripheral macrophages TMEV-IDD model, highlighting the contribution of M1/M2-liked phenotypic changes in microglia /macrophages to disease progression during TMEV infection.

## 2 Overview of TMEV

TMEV can be classified into two subgroups, based on neurovirulence: highly neurovirulent (George Davis 7–GDVII) and low neurovirulent (Theiler’s original—TO). The strains of GDVII and FA are contained within the GDVII subgroup. Among the strains in the TO subgroup are the DA, BeAn 8,386 (BeAn), TO4, Yale, WW, and 4,727 (Jarousse et al., 1998; Pavelko et al., 2012; Brinkmeyer-Langford et al., 2017; Sato et al., 2017). The highly neurovirulent strains can cause fatal encephalomyelitis in mice or mice die within 1–2 weeks of infection (Jarousse et al., 1998; Pavelko et al., 2012; Brinkmeyer-Langford et al., 2017; Sato et al., 2017). In contrast, the low neurovirulent strains cause different diseases depending on the mouse strain (DePaula-Silva et al., 2017). The intracerebral (i.c.) infection of Swiss Jim Lambert (SJL) mice with low neurovirulent strains, such as DA or BeAn, causes encephalitis during the acute stage of infection and demyelinating disease during the chronic stage of infection develops a MS-like disease termed TMEV-induced demyelinating disease (TMEV-IDD) (Daniels et al., 1952; Lipton, 1975; Libbey et al., 2008; Tsunoda and Fujinami, 2010), while the C57BL/6J mouse strain, when i.c. infected with TMEV, develops acute seizures that progress to epilepsy (Libbey et al., 2008). The difference between the mouse strains seems to be partially explained by the strong antiviral innate immune response, for example type I interferon (IFN) in C57BL/6J mice (Rodriguez et al., 1995).

The TMEV model is an important tool for the study of neuroinflammatory and neurodegenerative diseases, providing a key platform for investigating the pathophysiological mechanisms of the diseases and developing potential therapeutic strategies. The following sections will focus primarily on the TMEV-IDD model by describing the roles of microglia and macrophages.

## 3 MS

**Figure 1:**
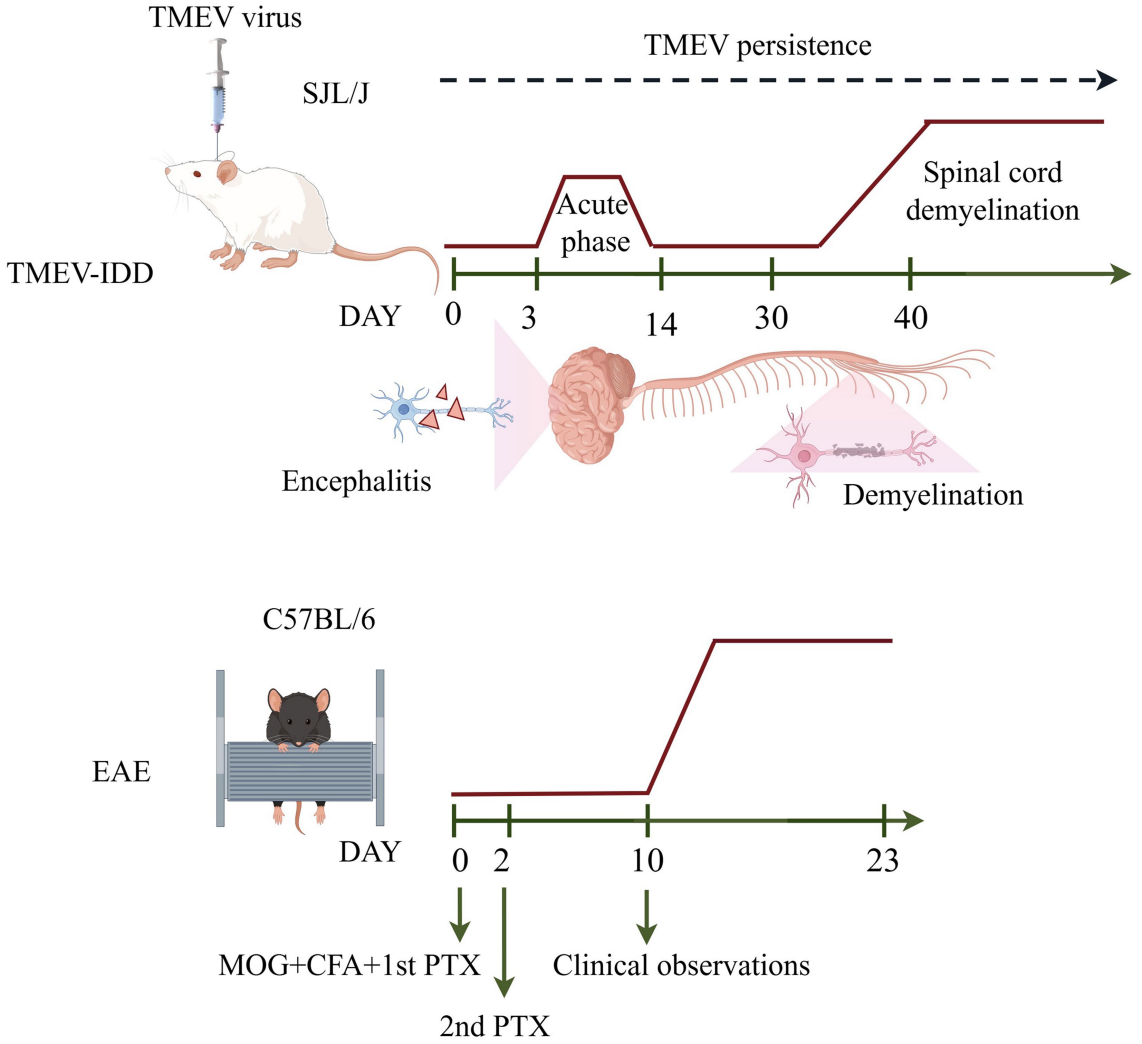
Schematic representation of mouse models of EAE and the TMEV-IDD.

MS is a cell-mediated chronic progressive neuroinflammatory and neurodegenerative autoimmune disease of the CNS characterized by inflammatory demyelination, axonal damage and progressive neurological dysfunction (DePaula-Silva, 2024). MS shows clear geographic variations, with higher rates in Europe and North America and lower rates in sub-Saharan Africa and East Asia, but its overall prevalence is increasing globally (Walton et al., 2020). MS usually develops between the age of 20 and 50 and is more frequently diagnosed in women (Rolak, 2003). As an autoimmune disease, the host immune system attacks its own myelin proteins. In individuals with MS, due to the myelin destruction, the saltatory conduction is impaired resulting in inefficient (Zhang et al., 2022). Signs and symptoms of the disease include cognitive and motor impairment, vertigo, loss of vision, weakness and dementia (Magyari, 2016; Psenicka et al., 2021). The pathogenesis of MS is not yet clear, but genetic and environmental factors may be strongly associated with the development of the disease (Davis et al., 2013). The experimental autoimmune encephalomyelitis (EAE) animal model and TMEV-IDD animal model are the most commonly used animal models for studying MS. The choice of the most precise model is predominantly influenced by the particular research and/or experimental question to be addressed. The main stages in the pathogenesis of EAE and TMEV-IDD are shown in Table 1, and schematic representation of mouse models of EAE and the TMEV-IDD are given in Figure 1.

**Table 1:** Characteristics of EAE and TMEV-IDD models of MS.

MS animal models	EAE	TMEV-IDD
Mouse species	SJL/J mice	C57BL/6 J mice	SJL/J mice
Induction methods	Immunization of SJL/J mice with PLP_139–151_	Immunization of C57BL/6 J mice with MOG_35–55_	Infection with TMEV of strain BeAn into SJL/J mice.
Pathogenic mechanism	Peripheral T cells are activated by viruses or another infectious antigens (similarity to some CNS antigens), these T cells are capable of producing inflammatory cytokines and have the potential to differentiate on activation into Th1 or Th17 cells or cells producing both. Activated T cells can cross the blood–brain barrier (BBB) by upregulating integrins, such as VLA-4. By penetrating the BBB, other immune cells including B cells and mononuclear/macrophages migrate to the CNS under the attraction of chemokine release. There, they encounter the homologous antigens, which are presented by resident or immigrant antigen presenting cells, such as, macrophages/microglia, dendritic cells or astrocytes. On encountering the antigen, these autoreactive T cells are reactivated, producing the cytokines, which activate neighboring immune cells or neural cells and attract further inflammatory cells into the CNS. Among them, activated macrophages are thought to indirectly or directly damage the CNS, promotes the demyelinating process.		CNS damage is initially mediated by antiviral responses, characterized by the recruitment of immune cells (primarily antiviral T cells) into the CNS. On the contrary, the chronic progression of the disease is mainly mediated by the activation of microglia, infiltration of macrophaes and mature B cells, which they release pro-inflammatory cytokines, leading to subsequent loss of myelin.
Type of corresponding MS	Relapsing–remitting MS	Primary progressive MS, secondary progressive MS	Primary progressive MS
Clinical significance	Study of neuroinflmmation and immune system activation and testing therapeutical agents.		Study of axonal damage and inflammatory-induced demyelination. Research on the new treatments targeting adhesion molecules, axonal degeneration.
Differences	Axonal damage occurs secondarily to severe inflammatory demyelination, where lesions develop from the periphery (myelin) to the inside (axon; outside-in model). Conversely, in TMEV infection, axonal damage precedes demyelination (inside-out model). Axonal degeneration triggers the recruitment of T cells and macrophages into the CNS, subsequently leading to demyelination.		
Reference	Whitham et al. (1991), McRae et al. (1995), Miyagawa et al. (2010), and Alhazzani et al. (2021)	Mendel et al. (1995), Bullard et al. (2007), Berard et al. (2010), Constantinescu et al. (2011), and Montilla et al. (2023)	Kim et al. (2005), Tsunoda and Fujinami (2010), Sato et al. (2011), Gilli et al. (2016), and Pike et al. (2022)

Recent research has revealed several promising strategies to improve the process of MS. For example, Bernardo-Faura et al. found that TAK1 inhibitors, in combination with existing MS drugs, significantly improved MS in an animal model of the disease, based on network-based modelling (Bernardo-Faura et al., 2021). Similarly, Kerstetter et al. discovered that the blockade of chemokine receptors, such as CXCR2, promotes the regeneration of myelin sheaths and enhances functional recovery in MS models (Kerstetter et al., 2009). Furthermore, mTOR inhibitors like rapamycin have demonstrated efficacy in reducing disease severity in MS models by balancing the immune response and promoting oligodendrocyte survival (Vakrakou et al., 2022). Another crucial pathway, the Keap1/Nrf2/ARE pathway, is essential for the regulation of oxidative stress and inflammation in MS (Michaličková et al., 2020), offering potential therapeutic targets for future interventions.

### 3.1 TMEV-IDD, an animal model of progressive MS

TMEV-IDD, which is a model for progressive forms of MS (Pike et al., 2022). TMEV-IDD in SJL/J mice is characterized by an acute phase, that occurs first week post-infection, which have high viral replication in neurons, and a chronic phase, which begins within 1 month after TMEV inoculation, marked by persistent infection in glial cells and chronic demyelination (Tsunoda and Fujinami, 2010). During the chronic phase of the disease, SJL/J mice infected with TMEV exhibit progressive weakness in their hind limbs, leading to severe spastic paralysis with no observed recovery, similar to what is observed in patients with the primary progressive multiple form of MS (Tsunoda and Fujinami, 2002; DePaula-Silva et al., 2017). In this disease model, the presence of demyelination in the CNS is associated with a prolonged inflammatory immune reaction caused by the persistence of the virus.

Toll Like Receptors (TLR) recognize pathogens through pathogen-associated molecular patterns (PAMPs), triggering the innate immune response and inducing pro-inflammatory chemokines and cytokines to recruit immune cells. Viral infections lead to the induction of type I IFN and IFN-γ cytokines. Activation of the type I IFN pathway increases levels of IFN-α and IFN-β and the type II IFN-γ. Activation of the type I IFN pathway leads to increased levels of expression of interferon-stimulated genes (ISGs), which can exert an antiviral effect (Biron, 2001; Olson and Miller, 2004; Olson, 2014; Bolívar et al., 2018).

In the acute phase, neurons within the hippocampus and cerebral cortex are predominantly infected (Gerhauser et al., 2019). Elevated levels of CD4^+^ and CD8^+^ T cells, monocytes, and a small number of B cells and plasma cells have been detected in the grey matter of the brain, indicative of CNS inflammation (encephalitis) (Frischer et al., 2009; Michel et al., 2015; DePaula-Silva et al., 2017; Faissner et al., 2019). Following the initial acute phase, a reduction in viral load occurs. However, the immune response is insufficient to fully eradicate the viral infection, resulting in progression to the chronic phase. During this chronic phase, inflammatory cells persist in the white matter while demyelination in the spinal cord and axonal damage are also observed (Martinat et al., 1999; Kummerfeld et al., 2012). Meanwhile, TMEV-infected SJL/J mice exibit a notable decrease in the quantity of neuronal progenitor cells and early postmitotic neurons in hippocampus at the chronic phase. Deficits in hippocampal neurogenesis observed following TMEV-infected SJL/J mice have mirrored the learning and memory impairments (Jafari et al., 2012). Unlike the acute phase, TMEV is not localized in neurons but instead remains present in oligodendrocytes, astrocytes, and microglia/macrophages, as evidenced by immunohistochemistry and *in situ* hybridization techniques (Clatch et al., 1990; Girard et al., 2002; Misra et al., 2008). Therefore, while TMEV persistence is important to induce demyelination, the trigger mechanism for demyelination is inflammation and the induction of autoimmunity.

## 4 Profile of microglia and macrophages

Microglia are the innate immune cells in the CNS, accounting for approximately 10–15% of all brain cells (Onose et al., 2022). They originate from myeloid lineage in the yolk sac and infiltrate into the CNS during embryonic development (Ginhoux et al., 2010). In contrast, peripheral macrophages/monocytes are derived from adult bone marrow hematopoietic stem cells, under pathological conditions, monocytes can infiltrate the CNS and differentiate into macrophages. Both microglia and macrophages can drive neuroinflammation and promote the development of MS (Zaslona et al., 2012; Jiang et al., 2022).

*In vitro* experiments have demonstrated that microglia can sustain a persistent infection with TMEV, enabling them to induce a pro-inflammatory state in distal, uninfected cells through the secretion of viral RNA-laden exosomes. These exosomes subsequently activate microglia, enhancing their ability to process viral and myelin epitopes and to prime memory CD4^+^ Th1 cells. Intriguingly, direct infection of microglia with TMEV proves almost as effective as high levels of IFN-γ stimulation in imparting antigen-presenting cell (APC) functionality (Olson et al., 2001; Luong and Olson, 2021). Highly purified macrophages, isolated from mice infected with TMEV, exhibit exceptionally potent APC capabilities, akin to those of microglia, particularly in the context of chronically induced demyelinating disease (Chastain et al., 2011).

Microglia exhibit many functional and phenotypic similarities to peripheral macrophage due to their common myeloid lineage, including the presence of similar surface molecules, which makes it difficult to distinguish the two populations of cells in the CNS during an immune response to TMEV, such as CD 11b. However, microglia can be distinguished from macrophage by their lower expression level of CD45 (Aloisi, 2001). In addition, DePaula-Silva et al. studied the functional distinctions and commonalities in the gene expression profiles of microglial and macrophage immune responses during neurotropic viral infection. Among these genes, 43 were found to be expressed exclusively by infiltrating macrophages, 43 were uniquely expressed by reactive microglia, and 50 were identified as being commonly expressed in both infiltrating macrophages and reactive microglia. They suggested the identified and validated genes that are uniquely expressed at the cell surface of microglia or macrophages, which can be used to distinguish between these two cell populations (DePaula-Silva et al., 2019).

### 4.1 The role of microglia and infiltrating macrophage in demyelination following TMEV CNS infection

The role of microglia/macrophages in TMEV-IDD is partially related to the ability of virus to persist in these cells. During the acute period, the numbers of activated microglia were increased in the brains of SJL/J mice after TMEV infection, which inhibited the survival of neural progenitor cells and impeded neuronal differentiation, ultimately leading to hippocampal neuropathy (Ekdahl et al., 2003; Monje et al., 2003; Jafari et al., 2012). Moreover, during demyelination, microglia/macrophages are found near the lesion site, which contain TMEV virus antigens. Microglia/macrophages are capable of efficiently engulfing viral particles through phagocytosis. Although studies have demonstrated persistent infection of microglia/macrophages by TMEV in SJL mice, these cells exhibit low permissiveness for viral replication, and produced few viral particles during TMEV infection (Rodriguez et al., 1983; Clatch et al., 1990; Lipton et al., 1995; Rossi et al., 1997; Trottier et al., 2001). Microglia are capable of detecting a viral infection or cell-associated damage signals through pattern recognition receptors (PRRs) and damage-associated molecular patterns (DAMPs), respectively, leading to their activation. Reactive inflammatory microglia trigger the expression of Type-I IFN (IFN-I), and NF-κB, thus up-regulating the expression of inflammatory mediators including IL-6, IL-12, tumor necrosis factor (TNF)α, CCL2, CCL3, and CCL5 (Olson et al., 2001; Olson and Miller, 2009; Kim, 2021; Luong and Olson, 2021). In addition, excessive or continuous activation of microglia/macrophages leads to local tissue damage/immunopathology in CNS (DePaula-Silva et al., 2017). Activated microglia increase the expression of MHC-I and MHC-II, and costimulatory molecules, enhancing their ability to present antigens to T cells (Schetters et al., 2017; Moseman et al., 2020; Goddery et al., 2021). Presentation of viral antigens to Th1 CD4^+^ T cells results in the secretion of chemokines by these T cells, thereby enhancing the recruitment of peripheral macrophages. The excess of CNS inflammation causes bystander myelin damage. In TMEV-infected mice, macrophages and microglia have the ability to uptake myelin antigens, then presenting them to autoreactive myelin-specific CD4^+^ T cells (DePaula-Silva et al., 2017).

Microglia and macrophage activation is an initial occurrence in MS/TMEV and persists throughout the course of the disease (Gentile et al., 2016; Bell et al., 2020). Sustained expression of M1-liked related genes in microglia/macrophage leads to persistent inflammation and hindered myelin regeneration (Herder et al., 2015). Furthermore, M1-liked microglia are known to secrete proteolytic enzymes, including matrix metalloproteinases (MMPs), which contribute to the degradation of myelin basic protein (MBP) and subsequent direct damage to myelin (Liuzzi et al., 1995; Ulrich et al., 2006). Hansmann et al. indicates that deficiency in MMP12 is linked to decreased activation of M1-liked microglia during the TMEV demyelinating phase, suggesting a significant involvement of MMP12 in the activation and polarization of microglia (Hansmann et al., 2019). Additionally, TMEV can trigger the activation of NLRP3 inflammasome through the Toll-like receptor (TLR) signaling pathway. NLRP3 inflammasome, function as signaling platforms for caspase-1-driven activation of IL-1-type cytokines (IL-1β and IL-18), which contributed to the neuroinflammatory response in the CNS (Tsunoda and Fujinami, 2010; Gerhauser et al., 2012; Jeong et al., 2022). Activation of the NLRP3 inflammasome and the downstream PGE2 promote the pathogenesis of TMEV-induced demyelinating disease by enhancing the production of IL-17 (Kim et al., 2017). Moreover, NLRP3 polarizes microglia/macrophage toward a pro-inflammatory M1-liked phenotype, promote microglia/macrophage-mediated neuroinflammation and contribute to neuroprogression (Wang et al., 2020; Yang et al., 2022). As discussed previously, M1-liked microglia/macrophage have detrimental effects in the course of MS/TMEV (Mikita et al., 2011; Guo et al., 2022). Nevertheless, it does not mean that pro-inflammatory mediators such as M1-liked microglia /macrophage and Th1 cells only contribute adversely to the pathogenesis of MS/EAE. Differentiation of appropriate numbers of M1-liked microglia have been shown to promote neurogenesis and oligogenesis, whereas excessive activation has been found to be inhibitory (Butovsky et al., 2006a,b). In contrast, M2-liked microglia/macrophage are regulatory/anti-inflammatory and secrete regulatory cytokines such as IL-10 and TGF-β, promoting tissue repair and resolving inflammation within the CNS by downregulating M1 and Th1 immune responses (Laskin, 2009; Herder et al., 2015). Park HJ et al. demonstrated the important contribution of M2-liked microglia/macrophage in the clearance of debris and support of neuronal survival by releasing neurotrophic factors and participating in phagocytosis (Oñate et al., 2016; Park et al., 2016).

It has been revealed that an imbalance of M1-liked cells in the spinal cord of infected mice (Miron et al., 2013; Colombo et al., 2014; Hansmann et al., 2019). In addition, TMEV preferentially infects activated myeloid cells that exhibit pro-inflammatory functions M1-like, which expressed CD16/32 and IFN-γ, *in vitro* (Jelachich and Lipton, 1999). It is also tempting to speculate that prolonged M1-liked polarization contributes to viral persistence in susceptible mouse strains by creating an environment conducive to the virus. With disease progression an accumulation of neuroprotective M2-liked cells was found. Despite mounting M2-liked polarization and the expression of regeneration promoting factors, such as Tgfb1, CNS recovery restricted and only insufficient remyelination attempts by oligodendrocytes and Schwann cells were found in the spinal cord during the late chronic TME phase (Herder et al., 2015). M2-liked microglia/macrophages were not reverse the long-term damage to the CNS caused by M1-liked microglia/macrophages in TMEV-IDD.

## 5 Drugs targeting microglia/macrophages

Various pharmacological interventions aimed at altering the behavior of microglia have demonstrated potential in the treatment of TMEV infection and MS by modulating immune responses, reducing inflammation, and promoting remyelination. Qie et al. discovered that candesartan, an AT1 receptor blocker (ARB), effectively regulated the neuroinflammatory response, reversed neurotoxic effects, and induced a shift in microglia from the M1-liked to M2-liked phenotype, at least in part through the inhibition of the TLR4/NF-κB signaling pathway (Qie et al., 2020). Edetomidine, an agonist of the alpha2 adrenoceptor, induces M2-liked microglia polarization through the inhibition of ERK1/2 signaling (Qiu et al., 2020). Shen et al. have engineered neutrophil-derived nanovesicles (NNV) to modulate neuroinflammation by enhancing the clearance of myelin debris in microglia (Shen et al., 2022). Additionally, Zheng et al. have developed a formulation called resveratrol-containing RAW-Exo (RSV&Exo) that targets microglia to mitigate neurodegeneration (Zheng et al., 2023). Mecha et al. found that the administration of the endogenous cannabinoid 2-AG selectively targets microglia, improving their capacity to clear myelin and promoting the differentiation of oligodendrocyte progenitor cells, thereby enhancing myelin regeneration (Mecha et al., 2019). In a MS animal model of EAE, lipoic acid was reported to reduce microglia/macrophages activation and the occurrence of MS disease (Kamma et al., 2022). It seems the mechanisms underlying microglia/macrophages polarization are complex, and combining different mechanisms together may have a helpful effect in the therapy of MS. Relationship between the pathogenesis of MS and microglia/macrophages polarization remains to be studied.

## Author contributions

QZ: Funding acquisition, Visualization, Writing – original draft, Writing – review & editing, Data curation. WS: Data curation, Visualization, Writing – review & editing, Formal analysis. MZ: Data curation, Formal analysis, Visualization, Writing – review & editing. NZ: Visualization, Writing – review & editing, Conceptualization, Funding acquisition, Project administration, Supervision, Writing – original draft.
